# New ways of looking at an old disease: the reimagination of epilepsy

**DOI:** 10.1007/s00415-016-8025-3

**Published:** 2016-02-04

**Authors:** Clinton J. Mitchell, Neil P. Robertson

**Affiliations:** Department of Neurology, Institute of Psychological Medicine and Clinical Neuroscience, Cardiff University, Cardiff, UK

The last 20 years have witnessed significant advances in the field of epilepsy with a host of new diagnostic descriptions and treatment options for the neurologist. In this month’s journal club, we review 3 papers which together provide a brief glimpse into an evolving innovative approach to epilepsy and also address some common dogmas in the nature and treatment of this common condition.

Contemporary classification of the epilepsies has introduced a new vocabulary to communicate between medical staff and patients. In parallel the International League Against Epilepsy (ILAE) has recently proposed a similar redefinition and reclassification of status epilepticus (SE) which is likely to have a significant effect on patient management and is the focus of the first paper reviewed. The second sets out to debunk one of the last held myths in epilepsy: that a generalised epilepsy syndrome (now often recognised to have a genetic cause) does not have associated architectural changes within the cortex. Finally, we examine recent evidence that perampanel may have a role in treating not only focal epilepsies but also in the adjunctive treatment of patients with refractory generalised seizures.

## A definition and classification of status epilepticus—report of the ILAE Task Force on Classification of Status Epilepticus

A standardised definition of SE has long evaded medical terminology with previous definitions relying on ambiguous phrases including a seizure as a “fixed and enduring condition”, or one persisting for a “sufficient length of time.” The authors of this paper have sought to provide a new definition of SE, with a stated goal of providing a structured terminology, but one which could evolve as advances in neurobiology and pathophysiology became apparent. The suggested terms consist of a time point (*t*_1_) after which time a given seizure is considered to be “an abnormally prolonged seizure”, and a second time point (*t*_2_) after which time neuronal injury is thought to occur. This second time point provides a degree of impetus about how aggressive one should be in treating an abnormally prolonged seizure. The values of these time points vary depending on underlying seizure type.

This new classification of SE involves four axes: the semiology of the seizure, aetiology, EEG correlate, and the age of the patient. The goals of reclassification are to improve communication between healthcare professionals, improve treatment of patients, permit epidemiological studies, and guide basic research.

*Comment.* This paper follows on from the natural success of the earlier redefinition and reclassification of the epilepsies from the ILAE. However, it is important to stress that the new definition, by their own admission, is based upon incomplete evidence, and “considerable variation”. Whilst they have allowed for the advent of future knowledge in the decision to include two time points, it may have been simpler to adopt a new single approach with the lowest common denominator. Classifying a generalised tonic–clonic seizure ‘abnormally prolonged’ after 5 min, but a focal motor seizure ‘prolonged’ after 10 min might appear pedantic since clinicians may not make this distinction in clinical practice. Furthermore, there may be concerns in the suggested guidance that a treating clinician has greater than 60 min before the onset of neuronal injury since it may promote a delay in treatment as a result of a perceived lack of urgency. Furthermore, in spite of these specific time frames, the authors acknowledge that the likelihood of damage is dependent on the location of the epileptic focus the intensity of the status, and the age of the patient.

The reclassification of SE provides a more reasoned argument. Certainly, improved communication between healthcare professionals is needed in the emergency setting. However, this also highlights a limitation since it is directed at an audience most accepting of the proposed changes. It could be argued that the optimum target for reclassification should be Emergency Physicians who are more likely to encounter SE at presentation. Nevertheless, this more accurate guide to the description, semiology and aetiology of the seizure will undoubtedly aid management. Less helpful in the acute management (but more so with ongoing management) is an EEG correlate, an investigation that may not always be provided in a timely fashion.

Trinka E et al. (2015) Epilepsia 56(10):1515–1523.

## Cortical microarchitecture changes in genetic epilepsy

The identification of genetic underpinnings of generalised epilepsy has allowed for the development of animal models of these epilepsies. This paper described the situation of a GABA_A_γ2(R43Q) mouse which replicates the human phenotype of absence seizures with spike-and-wave discharges, and reduced threshold to thermal seizures. A pedigree with a pure febrile seizure phenotype was chosen to determine the effect of this mutation on cortical development. A labelled, viral vector was inserted into the thalamus to aid in histological interpretation. A putative area of cortex was studied against a control area in the somatosensory cortex. The authors found an increased density of GABAergic neurons, but also that the ratio between excitatory and inhibitory GABAergic neurons was reduced (suggesting that inhibitory neuron density alone did not predict function). Additionally, changes in the diversity of inhibitory neurons were seen at different cortical layers, especially in layers associated with high levels of expression of the γ2 subunit during embryonic development. This paper suggested that microscopic changes are seen in specific areas of the cortex associated with a specific model of a genetic generalised epilepsy.

*Comment.* This paper is an important first step in the understanding of the effects of mutations on cortical structures, and provides a springboard for future efforts and replication. However, there are a number of limitations to the paper itself: Firstly the *SCN1A* mutation is the more well-characterised mutation but fails historically as a mouse model. Secondly, the GABA_A_γ2 mouse was used as its own control for neuron density and type. Thirdly, the area of cortex studied is not well developed in humans. It would have been a useful adjunct to this paper, if mouse MRI data were also included.

However, this paper does provide an interesting insight suggesting that it is not solely inhibitory neuron density that is responsible for changes in cortical excitability, but perhaps the ratio with excitatory neurons. This may provide an explanation of the differences seen in transcranial magnetic stimulation excitability in different epilepsies.

Wimmer VC et al. (2015) Neurology 84:1308–1316.

## Perampanel for tonic–clonic seizures in idiopathic generalised epilepsy—a randomised trial

This paper reports a well-designed, multicentre, randomised, placebo-controlled trial that demonstrates clear efficacy for perampanel in the adjunctive management of patients with refractory generalised epilepsy. The population group and range of current medications was similar to that in clinical practice. There was a 76.5 % reduction in median seizure frequency in the treatment group, with a greater than 50 % response rate seen in 64.2 % of the treatment group. Complete seizure freedom was achieved in 23.5 % of the treatment group. Adverse events (AE) were comparable between the two groups, and serious AE were only seen in a minority of patients, and were manageable with medication withdrawal. One death occurred in each group: SUDEP in the placebo arm, and an accidental drowning in the treatment arm. No biochemical abnormalities were noted in either group. This paper provided justification for inclusion of perampanel into the pharmacopoeia for refractory generalised epilepsy.

*Comment.* This paper has built on the previous success of three previous randomised, placebo-controlled trials in the management of focal epilepsy. The study was well designed but did include the inferior primary endpoint of percent change in seizure frequency per 28 days, over the more acceptable and widely used 50 % seizure responder rate (although this was included as a secondary endpoint for the purpose of registration according to European Union guidelines). A major strength of the study was the rigorous screening that patients went through to ensure they had idiopathic generalised epilepsy (now recognised as a genetic epilepsy) on the basis of age at onset, EEG data, MRI data, and seizure description. This allowed a much stronger effect size to be seen, which the authors point to when making comparisons with other benchmark trials of efficacy in generalised epilepsy.

The drug was well tolerated and no unexpected AE were identified. One SUDEP death amongst the placebo group continues to raise concerns about the design of trials in generalised epilepsy that have placebo arms, and how to protect these at-risk patients. Earlier trials of perampanel had allowed greater doses (up to 12 mg) that were not seen in this paper (maximum 8 mg). The reason for this was not stated.

The trial is now undergoing an open-label extension and the results of this are eagerly awaited.

French JA et al. (2015) Neurology 85:950–957.

